# Short, Animated Storytelling Video to Reduce Addiction Stigma in 13,500 Participants Across Multiple Countries Through an Online Approach: Protocol for a Randomized Controlled Trial

**DOI:** 10.2196/73382

**Published:** 2025-05-05

**Authors:** Maya Adam, Maxwell Klapow, Merlin Greuel, Misha Seeff, Julia K Rohr, Andrew Gordon, Doron Amsalem, Till Bärnighausen

**Affiliations:** 1 Department of Pediatrics Stanford University School of Medicine Stanford, CA United States; 2 Heidelberg Institute of Global Health University Hospital Heidelberg Heidelberg Germany; 3 Department of Medicine Center for Digital Health Stanford Medicine Stanford, CA United States; 4 Department of Experimental Psychology University of Oxford Oxford United Kingdom; 5 United World College Maastricht Maastricht The Netherlands; 6 Prolific Academic London United Kingdom; 7 Department of Psychiatry Vagelos College of Physicians and Surgeons Columbia University New York United States; 8 Department of Global Health and Population Harvard T. H Chan School of Public Health Cambridge, MA United States; 9 Africa Health Research Institute (AHRI) Somkhele, Kwazulu Natal South Africa

**Keywords:** stigma reduction, addiction, health communication, social media, animation, video, storytelling.

## Abstract

**Background:**

Stigma toward people with addiction is a well-documented phenomenon that dramatically impacts help-seeking, treatment, and recovery. Interventions aimed at reducing stigma toward those with addiction must overcome the frequent mischaracterization of addiction as a failure of judgment rather than a chronic, treatable illness. Previous research has demonstrated that social contact with people recovering from addiction can promote empathy and reduce stigma, but social contact is difficult to scale. Short, animated storytelling (SAS) is a novel health communication approach that scales easily because it can leapfrog barriers associated with language, culture, literacy, and education levels.

**Objective:**

This study will investigate the effect of a cross-culturally accessible SAS video intervention aimed at reducing stigma and increasing empathy toward people with addiction. We also seek to gain insight into the mechanisms of action of this SAS intervention by measuring the contribution of sound design to their effect.

**Methods:**

We will conduct a randomized controlled trial with 13,500 adult participants from the United States, the United Kingdom, and South Africa, recruited online via Prolific Academic and randomized into 3 arms, per country. The 2 intervention arms will receive a wordless, social contact–based SAS video, one arm with a soundtrack and one without. The third arm will receive an educational video about addiction. Validated questionnaires will be used to assess our primary outcome, addiction stigma, and secondary outcomes, optimism, warmth toward the subject, and hopefulness, at baseline, immediately post exposure, and 2 weeks later. Ethics clearance was obtained on August 15, 2024, from the Stanford University institutional review board (protocol 76457).

**Results:**

This trial was funded in January 2025 by the Heidelberg Institute of Global Health, the Faculty of Medicine at Heidelberg University, in Germany. As of March 2025, no data have been collected. The estimated start date for this trial is May 15, 2025. We expect to complete data collection by July 1, 2025, and expect results to be published in the spring of 2026.

**Conclusions:**

Here, we present the protocol for an online, multicountry, randomized controlled trial. This trial is designed to measure the effect of an innovative approach to global health communication (wordless, short, and animated storytelling) on addiction stigma in 3 global regions. These findings will inform the design of future scalable, digital health storytelling interventions for global audiences while exploring the capacity of SAS to shift public health attitudes and perceptions. Furthermore, if effective, the intervention described here could be disseminated broadly via social media and other online platforms.

**Trial Registration:**

ClinicalTrials.gov NCT06705205; https://clinicaltrials.gov/study/NCT06705205

**International Registered Report Identifier (IRRID):**

PRR1-10.2196/73382

## Introduction

Stigma toward people with addiction is a well-documented phenomenon that dramatically impacts recovery by discouraging help-seeking behavior, isolating those affected, and increasing the likelihood of shame-related comorbidities such as anxiety and depression [1]. Similar to stigma in other domains, stigma toward people with addiction involves prejudice and discrimination, leading to compounding sequelae such as avoidance, self-stigmatization, and failure to seek treatment [2]. These effects have been documented cross-culturally and globally, worsening an already challenging public health issue [1,2]. The World Health Organization estimates that approximately 400 million people aged 15 years and older live with alcohol use disorders globally [3]. In addition, an estimated 39.5 million people aged 15-64 years are affected by drug use disorders [4]. Recent research suggests that the COVID-19 pandemic dramatically exacerbated these problems, particularly in low- and middle-income countries [5]. In addition, the pandemic aggravated social isolation and loneliness, exacerbating self-stigmatization, especially for underresourced populations that are at a greater risk of addiction [6-8]. Stigma can thus significantly widen health disparities, underscoring the need for effective interventions to ameliorate it.

Reducing addiction stigma is challenging because addiction is often viewed as a failure of judgment and lack of morality rather than a chronic illness, with complex causes (genetic and environmental) that require both medical and psychological intervention. Interventions aimed at reducing public stigma toward those with addiction must address this frequent mischaracterization of addiction [9]. Evidence of stigma has even been documented among health care professionals with observed negative impacts on quality of care [10]. Previous research has demonstrated that social contact with people recovering from substance use disorders can promote empathy and reduce stigma, but these interventions often require extended face-to-face interaction and are difficult to scale [11]. Intergroup social contact interventions, despite their promise for reducing stigma, can also be limited by individual selection bias, due to the very prejudice the intervention aims to reduce [12]. A further challenge lies in the fact that public health communication interventions often result in the systematic exclusion of non–English speakers and non-Western cultural groups [13]. Even scalable virtual interventions for stigma reduction and empathy building have produced mixed findings beyond high-income and homogeneous populations [14]. As a result, there is an urgent need for novel approaches that focus on broadly disseminating accessible, positive stories related to addiction to promote empathy and understanding. The major challenge lies in making them accessible across languages, cultures, education, and literacy levels.

Short, animated storytelling (SAS) is a novel health communication approach developed in response to the need for rapid, global dissemination of public health messaging across various languages, cultures, literacy, and education levels during the COVID-19 pandemic [15,16]. SAS interventions boost cross-cultural relevance through accessible character designs [17], and they support the engagement of diverse audiences through the use of wordless visual storytelling and compelling soundtracks [18,19]. These interventions are specifically designed for online environments, namely social media platforms, where people readily consume short video content and increasingly seek health information [20]. Social media has become a critical tool for public health communication, with a growing body of research evaluating these interventions [21]. The global reach of many social media platforms enhances their potential utility for scaling health communication interventions, including those aimed at reducing addiction stigma. However, such interventions must be rigorously tested in different global regions and their mechanisms of action must be better understood in order to inform the ongoing development of effective, scalable, and cross-culturally accessible interventions.

This study will investigate the effect of a globally accessible SAS video intervention aimed at reducing stigma and increasing empathy toward people with addiction. In addition, we will attempt to isolate and quantify the contribution of sound design to the effect of the intervention. This wordless, animated SAS intervention was co-created with a multidisciplinary team of experts who are trained in health communication, addiction medicine, animation, sound design, screenwriting, and digital storytelling. The video was designed to resonate globally, across cultures, and traverse linguistic, literacy, and educational barriers. We hypothesize that a single exposure to the 2.5-minute intervention video will measurably reduce addiction stigma and boost hope, optimism, and empathy toward people with addiction in three culturally distinct global settings: the United States, the United Kingdom, and South Africa. We also hypothesize that this effect will persist at a 2-week follow-up and that sound design will enhance the magnitude and durability of the observed effect.

## Methods

### Study Setting, Participants, and Recruitment Procedure

This study will be conducted online. Adult participants from the United States, the United Kingdom, and South Africa will be recruited and randomized through the platform Prolific Academic (ProA). These 3 countries were chosen because Prolific has a large enough number of participants in each of these countries to make it feasible to reach our desired sample size. The video intervention and questionnaires will be delivered to participants through the secure, online survey platform, Qualtrics, an experiment builder that provides users with the tools for undertaking online behavioral research.

We will enroll 4500 participants in the United States, 4500 participants in the United Kingdom and 4500 participants in South Africa using ProA, a crowdsourcing platform frequently used to conduct rigorous academic research [22]. Participants in this study will be adults aged 18-49 years. This age group is selected because they reflect our target audience: adults who are likely to seek information and regularly engage on social media [23]. Broad eligibility criteria reflect our interest in evaluating the intervention as it would be delivered outside of experimental conditions: online, widely available via social media or the Internet, and not targeted toward any specific audience. Before enrolling in the study, all participants will read an information sheet on ProA that has been approved by the Stanford Ethics Committee. Those who agree to participate will be directed by Prolific to complete the study activities on Qualtrics.com, a secure online data-collection platform.

### Randomization, Consent, and Study Design

After reviewing the Institutional Review Board-approved information sheet, delivered to prospective participants online via ProA, those who indicate their consent to take part in the study will then be randomly assigned to one of three groups:

1. Intervention A: receiving the SAS video full intervention group.
2. Intervention B: receiving the SAS video partial intervention (without sound) group.
3. Control Group: receiving written information about addiction prevalence.

A baseline survey will be used to collect demographic information as well as baseline data on our primary and secondary outcomes. Thereafter, the Intervention A group will watch the 2.5-minute video, the Intervention B group will watch the 2.5-minute video without sound, and the control group will read a fact sheet about the global burden of addiction. Following this, all groups will complete a series of validated questionnaires to assess our primary outcome: addiction stigma, and secondary outcomes: optimism, warmth toward the subject, and hopefulness.

**Figure 1:**
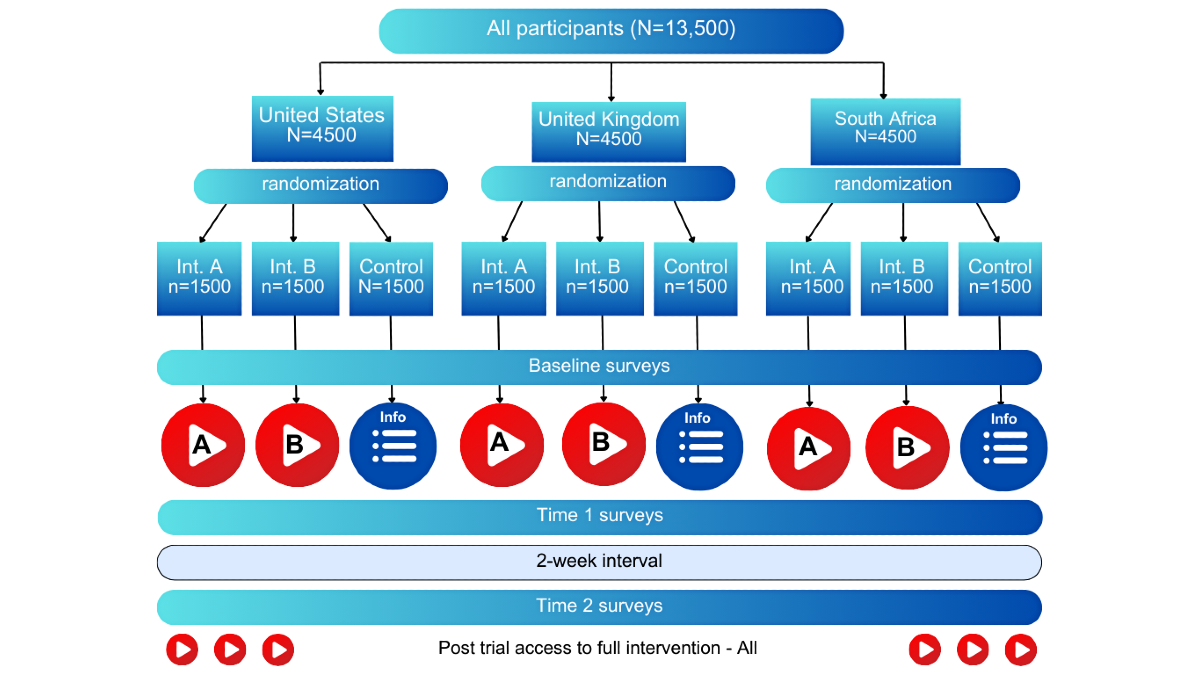
Trial design and flow.

We will conduct the first round of surveys immediately post exposure of the intervention groups to the SAS video interventions and the control group to their written fact sheet. Follow-up assessments, using the same surveys, will be conducted 2 weeks later. To ensure the accuracy and validity of the results, we will include attention checks and exclude participants who fail these checks. Each participant will spend approximately 8 minutes watching the intervention and completing the surveys and will receive US $4.80 for completing the study. Figure 1 represents the trial design and flow.

### Intervention

**Figure 2:**
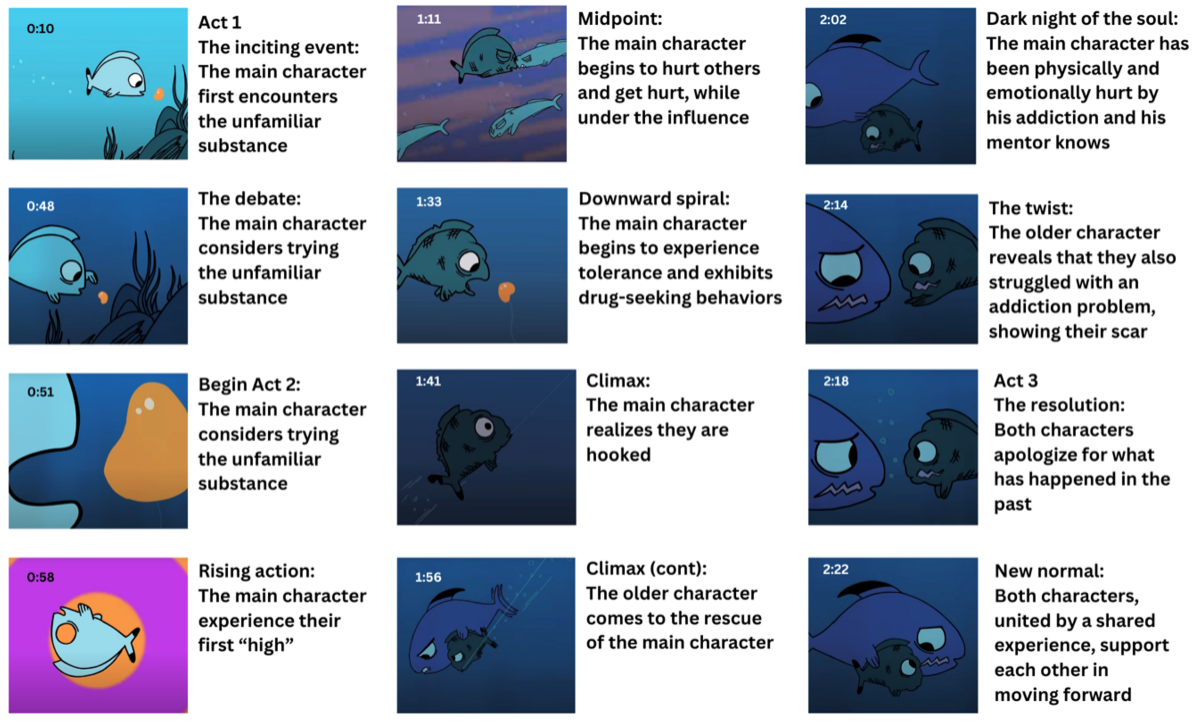
Selected scenes from short, animated storytelling intervention with timestamps.

This short, animated story video was co-created by a global health communication specialist with formal training in screenwriting and story development, an addiction medicine specialist, a professional animator living in South Africa, an adolescent composer and sound designer, and a group of high school student advisors at Avenues the World School in New York City. The intervention follows a classical 3-act story structure, with an inciting event, rising action, a midpoint, climax, and resolution. Figure 2 displays selected scenes from the SAS intervention video, with corresponding timestamps and descriptions of the story “beats” that propel the narrative forward. The main character is a fish who discovers and tries a foreign substance, ignoring the warnings of a more senior fish character. The young fish soon becomes addicted to the substance, and we see the negative impact on his underwater life. He injures others around him while under the influence and, finally, injures himself in his desperate pursuit of the next high. In this way, the video visually conveys the concept of drug tolerance. In the second half of Act 2, the main character, in desperation, gulps down the remaining dose of the addictive substance before realizing that he is (literally) hooked. A fishing line drags him off-screen and it seems that all is lost until the senior fish reappears and helps him break free of the hook. This leaves a deep gash in the young fish’s mouth. In the young fish’s moment of deepest pain and shame, the senior fish reveals his identical gash (the unexpected story twist). The 2 fish share a moment of painful empathy, then swim off-screen together. The only words in this video appear as text on the final, “call-to-action” screen: “Hooked on something that’s hurting you? Speak up. There’s help.” The video can be viewed on YouTube [24].

### Primary Outcome

The primary outcome is stigma toward people with addiction, which we will measure using an abbreviated, 18-item version of the Attribution Questionnaire (AQ-18) [25,26]. This version (AQ-18) includes the blame, pity, help, dangerousness, fear, and avoidance subscales of the original questionnaire, as these subscales are most appropriate, in scope and topic area, to detect a potential effect of our short video “micro-intervention.” The AQ-18 will be scored along a 9-point Likert scale indicating the extent to which participants agree with the item ranging from “not at all” (1) to “very much” to (9) with a maximum score of 27 for each 3-item construct. Higher scores indicate greater stigma. The AQ-18 is highly reliable and subscales have Cronbach *α* ranging from fair (0.6, responsibility) to good (0.93, fear/dangerousness) [25,26]. Participants responding to the AQ-18 may score between 12 and 108. Like the longer version, the AQ-18 offers a brief vignette, which we modified to be (1) gender-neutral and (2) about addiction rather than general mental illness. All participants will read the following vignette before completing the questionnaire. A gender-neutral name (Alex) was chosen to eliminate potential gender bias:

Alex is a 30-year-old with a history of addiction. Alex’s substance use problem has harmed Alex’s health and hurt others. Alex has been hospitalized several times for addiction-related health problems.

### Secondary Outcomes

#### Optimism

We will measure optimism using the Brief García’s Interactive Optimism Scale (BIOS-G) [27]. The BIOS-G is an instrument designed to assess an individual’s level of general optimism toward their life and other people. The scale includes 4 statements for which respondents indicate their level of agreement from 1 (“Of course not”) to 4 (“Yes, of course”). Higher scores indicate a greater level of optimism. Participants may score between 4, (indicating low optimism) and 16, (indicating high optimism). The BIOS-G has demonstrated reliability and validity across diverse populations, with a reported Cronbach *α* of 0.86 [27].

#### Attitude

We will also measure attitudes toward those with addiction using a “stigma thermometer,” a tool we previously developed to assess attitudes regarding sexual orientation and gender diversity [28]. The thermometer provides the following prompt:

Using a scale from zero to 100, please tell us about your personal feelings toward people who are addicted to substances such as drugs or alcohol. As you do this task, think of an imaginary thermometer. The warmer or more favorable you feel toward people who are addicted to substances such as drugs or alcohol, the higher the number you should give it. The colder or less favorable you feel, the lower the number. If you feel neither warm nor cold toward people who are addicted to substances such as drugs or alcohol, rate it 50.

Researchers in the fields of political science and psychology have frequently used feeling thermometers to assess attitudes toward stigmatized groups [29].

Finally, we will measure subjective levels of hope using a visual analog scale (VAS), a longstanding, validated tool for assessing related constructs of stress and subjective well-being [30]. The VAS provides the following prompt:

Using a scale from zero to 100, please tell us about how hopeful you feel after watching the video [control group participant instructions will reference the fact sheet in the AQ-27 instead]. As you do this task, think of an imaginary thermometer. The more hopeful you feel, the higher the number you should give it. The less hopeful you feel, the lower the number. If you feel neither hopeful nor hopeless, rate it 50.

#### Analysis

At the first time point (T1), we will compare addiction stigma, optimism, hope, and warmth toward subject scores between the 2 intervention groups and the control group. This comparison is intended to establish the immediate effect of watching the intervention videos on addiction stigma, optimism, hope, and warmth toward people with addiction. At the second time point (T2), we will once again compare our primary and secondary outcomes between the intervention and control groups. This will allow us to detect potential short-term effects of watching the intervention video on addiction stigma, optimism, hope, and warmth toward people with addiction. By comparing the outcomes of the 2 intervention arms at the 2 time points, we will be able to measure the contribution of sound design to the effect of the SAS video intervention.

#### Sample Size

We calculated the sample size needed for comparing mean scores between the 3 arms, as detailed in the trial design. This was performed independently for the primary and secondary outcomes based on the score of each survey, upon which we selected the most conservative sample size for our study.

We assume that a significant, sustainable change in addiction stigma from watching the SAS video could be achieved by multiple exposures to the video or similar SAS interventions in a series of “micro-doses.” As our study measures the effect of a single exposure to a single SAS video, we aim to measure a small change in our mean outcome scores, presuming that these small changes could yield larger ones following repeated exposures (microdoses). Thus, we have powered this trial to detect a small effect size for comparing the mean scores between trial arms, that is, *d*=0.1, smaller than the generally recommended threshold for detecting a small effect size [31].

We used the sample size formula for a 1-tailed comparison of the mean scores with equal variance, a Type I error of 0.01 to account for multiple comparisons, and a power of 80% to detect changes in the mean scores. This resulted in a sample size of 1128 individuals per arm. Accounting for a potential attrition rate of 20% (personal communication with ProA), this yields 1410 individuals per arm. For this study, we will recruit 1500 participants per arm in each of the 3 countries. This gives us a total sample size of 13,500 participants, which allows us to detect the potential effects of the intervention with a power higher than 80%.

#### Recruitment and Strategies to Improve Adherence to Interventions, Promote Participant Retention, and Complete Follow-Up

Reaching adequate participant enrollment for our target sample size relies on our choice of ProA as a recruitment platform. ProA has a sufficiently large database of registered prospective trial participants in the United States, the United Kingdom, and South Africa. To improve survey completion rates in this trial, we offer payment to all participants. Participants will receive half of their payment after Step 1 of the study and the remaining half after completing Step 2 of the study. If participants spend more than 45 minutes on any phase of the trial, they will be timed out of the experiment. This prevents participants from bottlenecking the survey platform with partially completed questionnaires. We also conducted a pilot trial with 600 participants in the United States only to verify participant retention. In this pilot study, 88% (526/598) of participants returned to complete the follow-up surveys, underscoring the feasibility of this trial [32].

#### Sequence Generation, Allocation Concealment, and Implementation

This 3-arm individual, parallel-group randomized controlled online trial will be hosted and implemented by the Qualtrics survey platform. The platform uses a computer-generated allocation sequence to randomly allocate participants 1:1:1 to the 3 trial arms. The investigators will remain unaware of individual trial arm allocations.

#### Blinding

All co-investigators and researchers involved in the data analyses will remain blinded to the trial arm allocation for the entire duration of the study. Statisticians will also be blinded to the study hypotheses, minimizing the risk of bias.

#### Participant Timeline

Participants will be enrolled over a period of 4 weeks via the ProA platform. Step 1 of the trial (baseline data collection, video viewing, and T1 surveys) will occur on a rolling basis, with Phase 2 (follow-up surveys) taking place 2 weeks later across all arms. After both study phases have been completed, all participants will be offered posttrial access to the intervention video. Their voluntary view times will be recorded as a measure of participant engagement with this health education medium (Table 1).

**Table 1:** Schedule of enrollment, interventions, and assessments.

Trial Information			Enrollment			Allocation			Close-out								
Time point			–t_1_			0			Intervention videos (t_1_)		Surveys (t_1_)		2-week interval		Surveys (t_2_)		Posttrial SAS^a^ video access
**Enrollment procedure**																	
	Eligibility screen	✓															
	Informed consent	✓															
	Allocation				✓												
**Intervention and assessments by arm**																	
	Intervention arms							✓		✓		✓		✓		✓	
	Control arm									✓		✓		✓		✓	

^a^SAS: short, animated storytelling.

#### Relevant Concomitant Care Permitted or Prohibited During the Trial

There is no relevant concomitant care, permitted or prohibited during this trial.

#### Criteria for Discontinuing or Modifying Allocated Interventions

Participants can withdraw their consent to participate at any time, before or during the study. Incomplete survey data will not be used in the primary analysis, except at a meta-level to report the aggregate number of incomplete surveys.

#### Provisions for Posttrial Care

This trial involves the viewing of a short, animated storytelling video featuring fish, after which a collection of validated surveys will be completed by participants in both groups. The trial will be conducted purely online.

There are no foreseeable risks associated with participating in this study. Participants volunteer and consent to participate in the study and are aware that they can withdraw at any time. The study investigators can be contacted at any time after the study, should the participants wish to follow up.

#### Plans for Assessment and Collection of Outcomes

The Qualtrics platform will collect data submitted by individual participants as they select their responses to the surveys used in this trial. We anticipate completing all data collection within a 6-week period.

#### Data Management

Qualtrics uses Transport Layer Security encryption for transmitted data. Qualtrics’ services are hosted by trusted data centers that are independently audited using the industry-standard Statement on Standards for Attestation Engagements 18 (SSAE-18) method. The research team conducting this study can access only anonymized data and, for statistical analysis purposes, we will download and safely store this data on secure computers maintained by our coinvestigators.

#### Statistical Methods for Analyzing Primary and Secondary Outcomes

##### Descriptive Statistics

We will use descriptive statistics to summarize the distribution of our outcomes by demographic factors, including age, gender, race/ethnicity, political affiliation, socioeconomic status, and previous experience with both addiction and childhood trauma, since these factors could affect scores.

##### Primary Outcomes

For each participant, stigma scores will be calculated individually. The mean score for each study arm at T1 and T2 will be calculated and compared between the intervention arms and the control arm at T1, and between the intervention arms and the control arm at T2, using repeated measure ANOVAs. This will be done at the study level and the country level to identify differential effectiveness across geographic conditions. We will also use multilevel regression analyses to control for demographic factors and other potential variables as needed. All analyses will be conducted in R (R Core Team, 2021).

### Secondary Outcomes

For the evaluation of secondary outcomes, we will use the statistical methods described above to evaluate both the immediate and medium-term effects of the SAS video intervention. The methods include, but are not limited to, *t* tests for independent groups, matched *t* tests for dependent groups, repeated measure ANOVAs, and multilevel regression models. For that, we will analyze the mean scores of the stigma surveys at T1 and T2, with a focus on the impact of the SAS video intervention on the score outcomes. All analyses will be conducted in R.

#### Methods for Additional Analyses (eg, Subgroup Analyses)

Exploratory subgroup analyses will be conducted to understand the extent to which demographic characteristics and country location moderate the effects of the intervention on stigmatization and secondary outcomes.

We will also use regression models to describe the potential effects of demographic factors on participant engagement. To quantify this engagement, we will use Qualtrics to offer participants voluntary posttrial access to the SAS video intervention. We will record a timestamp when the participant reaches the end of the second time point, the moment when a participant chooses to play the video and the moment when they end the study. Participants who skip the video or watch it for less than 3 seconds will be considered as not having engaged with the video. For the participants who watch the video, we will record the length of time spent watching it.

#### Methods in Analysis to Handle Protocol Nonadherence and Any Statistical Methods to Handle Missing Data

Participants in the study will have 45 minutes to watch the short, animated storytelling video and answer their surveys. All items of each survey are designed such that individual items must be answered before moving forward. This will guarantee that there will be no missing values in the datasets of participants included in the analyses. All participants who have completed the first part of the study (T1) will be included in the second part (T2). If a participant, who submitted a completed survey at T1, fails to complete a survey at T2, their data will be included only in the immediate effect analysis. Participants who do not watch the videos or answer the questionnaire items at either time point will be excluded from the analysis.

#### Access to Data

All researchers listed as co-authors on this study will have access to the full dataset. We have no contractual agreements with any other parties regarding the collection, evaluation, or use of the data collected as part of this study.

#### Data Monitoring and Trial Steering Committees

A trial steering committee (TSC) made up of an independent chairperson, additional members, and study collaborators will oversee this trial. A total of 2 TSC meetings will be conducted during the planning and data collection periods. Because this is a minimal-risk study with a very short study period, no additional data monitoring committee will be required. For the same reason, no interim analyses are planned, and no specific stopping rules are needed.

#### Adverse Event Reporting and Harms

This is a minimal-risk study. We do not anticipate any adverse events. The TSC will monitor study conduct during data collection. When providing consent, participants will be given a way to report adverse events should they occur during the trial.

#### Dissemination Plans

We anticipate the data collection to be completed by the end of July 2025. We aim to publish our findings in peer-reviewed academic journals, in the Spring of 2026, as well as presenting these at scientific conferences. Authorship will be considered according to the International Committee of Medical Journal Editors (ICMJE) criteria.

#### Plans to Give Access to the Full Protocol, Participant Level-Data, and Statistical Code

This document is the full protocol. Individuals interested in additional information or documentation should contact the corresponding author. The R code for the analyses will be published together with the planned publications on Github.com, as appropriate.

### Ethical Considerations

This study will be performed according to the ethical standards laid down in the 1964 Declaration of Helsinki and has been approved by the Stanford University Institutional Review Board (Protocol number 76457, approved on 8/15/2024.) We will follow the CONSORT (Consolidated Standards of Reporting Trials) guidelines [33] and the study has been registered in ClinicalTrials.gov (NCT06705205).

## Results

This trial was funded in January 2025 by the Heidelberg Institute of Global Health, at the Heidelberg University Faculty of Medicine, in Germany. As of March 2025, no data have been collected. The projected start date for this trial is May 15, 2025. We expect to have completed data collection by July 1, 2025, and we expect results to be published in the Spring of 2026. This is version 1.0 of the trial protocol and recruitment has not started for this trial. We anticipate beginning to recruit participants in March 2025. We anticipate that recruitment will be completed by May 1, 2025.

## Discussion

Here, we present the protocol for an online, multicountry, randomized controlled trial. This trial is designed to measure the effect of an innovative approach to global health communication (wordless, short, animated storytelling) on addiction stigma in 3 diverse global regions. Our hypothesis is that the SAS intervention tested here will measurably reduce stigma toward people with addiction, boost optimism, and improve attitudes toward people with addiction in 3 countries. We also hypothesize that the soundtrack will augment the effects of the intervention and will enhance the durability of those effects. Societal stigma toward individuals with addiction reduces help-seeking and impedes treatment and recovery from an increasingly prevalent global public health problem. During the design of this trial, we carefully considered the following potential limitations of our study. First, because not all countries have adequate numbers of potential participants enrolled on Prolific, and because our outcome measures are only validated in English, we were limited to three countries, in only three global regions: the United States, the United Kingdom, and South Africa. This subset is not representative of all global regions, so we will need to interpret our findings with caution, without overstating them. However, if our findings are promising, future studies could explore the effect of this intervention in other global regions, for example, in Asian countries where patterns and attitudes toward drug addiction differ from the global regions examined here [34]. Second, we acknowledge that our participant population may not be entirely representative of the general population in each country. Previous studies have shown that people registered on ProA tend to be younger and more educated than country averages [35]. However, the benefits of using ProA include providing concurrent access to large, geographically dispersed, adult test populations in different global regions, with minimal loss to follow-up [35].

### Conclusion

The findings can help establish future approaches to using short, animated storytelling interventions in cross-cultural settings, as well as characterize the extent to which these interventions can affect attitudes and perceptions. Furthermore, if effective, the intervention described here would be delivered via social media and other online platforms; therefore, we suggest that testing it with an online community is a reasonable approach for gauging its true effectiveness.

## Appendix

Multimedia Appendix 1 SPIRIT Checklist.

## Abbreviations

AQ-18: Attribution Questionnaire
BIOS-G: Brief García’s Interactive Optimism Scale
CONSORT: Consolidated Standards of Reporting Trials
ICMJE: International Committee of Medical Journal Editors
ProA: Prolific Academic
SAS: Short, animated storytelling
SSAE-18: Standards for Attestation Engagements 18
TSC: Trial Steering Committee
VAS: visual analog scale

## Data Availability

The datasets generated or analyzed during this study are available from the corresponding author on reasonable request.
